# Copper (II) Ion-Modified Gold Nanoclusters as Peroxidase Mimetics for the Colorimetric Detection of Pyrophosphate

**DOI:** 10.3390/s21165538

**Published:** 2021-08-17

**Authors:** Yunjing Shi, Jinjie Wang, Kun Mu, Suqin Liu, Guang Yang, Min Zhang, Jingxia Yang

**Affiliations:** 1 College of Chemistry and Chemical Engineering, Shanghai University of Engineering Science, 333 Longteng Road, Shanghai 201620, China; m040118166@sues.edu.cn (Y.S.); mkofficial@163.com (K.M.); m040119510@sues.edu.cn (S.L.); zhangmin@sues.edu.cn (M.Z.); jxyang@sues.edu.cn (J.Y.); 2 College of Chemistry, Chemical Engineering and Biotechnology, Donghua University, 2999 North Renmin Road, Shanghai 201620, China; gyang@dhu.edu.cn

**Keywords:** gold nanocluster, copper ion, pyrophosphate, keratin, colorimetric detection

## Abstract

Copper (II) ions have been shown to greatly improve the chemical stability and peroxidase-like activity of gold nanoclusters (AuNCs). Since the affinity between Cu^2+^ and pyrophosphate (PPi) is higher than that between Cu^2+^ and AuNCs, the catalytic activity of AuNCs-Cu^2+^ decreases with the introduction of PPi. Based on this principle, a new colorimetric detection method of PPi with high sensitivity and selectivity was developed by using AuNCs-Cu^2+^ as a probe. Under optimized conditions, the detection limit of PPi was 0.49 nM with a linear range of 0.51 to 30,000 nM. The sensitivity of the method was three orders of magnitude higher than that of a fluorescence method using AuNCs-Cu^2+^ as the probe. Finally, the AuNCs-Cu^2+^ system was successfully applied to directly determine the concentration of PPi in human urine samples.

## 1. Introduction

Pyrophosphate (PPi) is a byproduct of the hydrolysis of nucleoside triphosphates and plays an important role in many biological reactions [1]. According to previous studies, the level of PPi in human urine or synovial fluid has been associated with certain diseases, including chondrocalcinosis, urolithiasis, and even cancer [2,3]. PPi has been shown to inhibit the formation of calcium oxalate and calcium phosphate crystals in the urethra [4]. Therefore, the detection of PPi levels in urine is an important indicator for diagnosis and monitoring of urolithiasis. In the past few decades, many efforts have been made to develop methods for the quantitative detection of PPi.

Until now, a variety of different techniques have been devoted to conducting PPi detection, including fluorescence spectroscopy [5,6,7], surface-enhanced Raman-scattering (SERS) assays [8], colorimetric sensing [9,10,11], chemiluminescence [12], electrochemical analysis [13,14], and dark-field optical microscopy (DFM) [3]. The principle of most of the above methods is based on the complexation of PPi with metal ions (Al^3+^ [15], Fe^3+^ [9,16], Ca^2+^ [11], Zn^2+^ [17], and Cu^2+^ [5,6,10,12,13,18]), as PPi is a good complexing agent. From the above, fluorescence spectroscopy and colorimetric sensing are relatively common methods for detecting PPi. In addition, the fluorescence method has higher sensitivity than the colorimetric method and can be used for in vivo detection [19]. However, when comparing in vitro detection, the colorimetric method has the advantages of a fast detection speed, low cost, and convenient operation [20,21]. Therefore, it is still necessary to develop a simple and highly sensitive colorimetric analysis approach for PPi.

Many studies have shown that there is an interaction between Cu^2+^ and Au, and the affinity of this effect is lower than that between Cu^2+^ and PPi [22]. Based on these findings, a series of PPi detection methods have been developed.

Based on the principle that PPi can prevent Cu^2+^ from interacting with gold nanoparticles (AuNPs) to produce signal changes, Xiao’s team used AuNPs as probes to develop a color-coded single-particle detection method for PPi detection. The limit of detection (LOD) of this method was as low as 1.49 nM, and the detection range was 0–4.29 μM [3]. Tang’s group successfully synthesized Ag@SiO_2_-AuNCs as a core–shell composite nanostructure with metal-enhanced fluorescence (MEF) properties [23]. The fluorescence signal of the nanomaterial could be recovered by PPi after being quenched by Cu^2+^. The detection limit for PPi was 78.7 nM using Ag@SiO_2_-AuNCs as a sensor. Liu et al. reported a sensitive fluorescence quantification of PPi using BSA-AuNCs and a Cu^2+^ ion system. The detection limit of PPi was 83 nM with a linear range from 0.16 to 78.1 μM [24]. Our previous research showed that copper ions could not only reduce the fluorescence of AuNCs but also improve the chemical stability and peroxidase-like activity of AuNCs [25]. In addition, the concentration of Cu^2+^ had great influence on the increase in the catalytic performance of the AuNCs. Combined with the above studies on the interaction between Au, Cu^2+^, and PPi, we tried to develop a highly sensitive and selective colorimetric method to detect PPi.

In this study, a PPi colorimetric assay at 452 nm was developed using AuNCs-Cu^2+^ as a probe. As a control, a PPi fluorescence assay at 675 nm was also developed using the same probe. The reaction conditions of these two systems were optimized. The results showed that the LOD of the colorimetric method was much lower than that of the fluorescent method under optimized conditions. Furthermore, the principle and practical applicability of this colorimetric method were investigated.

## 2. Experimental Section

### 2.1. Materials

Gold chloride trihydrate (HAuCl_4_·3H_2_O, 99.9%), 3,3′5,5′-tetramethylbenzldine (TMB), and adenosine 5-triphosphate (ATP) disodium salt were supplied by Aladdin Chemistry Co. Ltd., (Shanghai, China). Sodium pyrophosphate was supplied by Sangon Biotech (Shanghai, China). H_2_O_2_ and Cu(NO_3_)_2_·3H_2_O were obtained from LingFeng Chemical Reagent Co. Ltd., (Shanghai, China). Keratins were prepared via reductive extraction [26]. Duck feathers were obtained from Sleep Hometex Co. Ltd., (Hangzhou, China). Other reagents were supplied by Shanghai Chemical Reagent Company.

### 2.2. Preparation of AuNCs-Cu^2+^

AuNCs were synthesized using keratin as the template according to our previously described method [25] and purified by filtering with a Sephadex G25 size exclusion column (SEC).

AuNCs-Cu^2+^ was prepared by simply mixing AuNCs with Cu^2+^ ions at room temperature. The optimal parameters for Cu^2+^ ion modification of AuNCs were 40 μM Cu^2+^ corresponding to 1 mg·mL^−1^ AuNCs. The stability of AuNCs-Cu^2+^ can be maintained for no less than 2 months when stored at 4 °C.

### 2.3. Colorimetric Assay of PPi

The colorimetric assay of PPi was based on the change in the peroxidase-like activity of AuNCs-Cu^2+^. PPi detection was performed according to a previously described method [25], with modifications: First, the samples (AuNCs-Cu^2+^, TMB, H_2_O_2_, and PPi) were mixed with Britton–Robinson (BR) buffer and incubated at a certain temperature for a period of time. The total volume of the reaction system was 900 μL. Second, the reaction solution was centrifuged at 12,000 rpm for 1 min to retain the precipitate. Afterwards, 300 μL of 2 M H_2_SO_4_ was added to the test tube. At this time, blue ox1-TMB adsorbed on the AuNC precipitate was converted into yellow ox2-TMB and dissolved in the solution. Finally, the absorbance of the supernatant obtained after centrifugation at 452 nm was detected by a UV-1601PC spectrophotometer (Shimadzu). The centrifugation and addition of H_2_SO_4_ could greatly improve the sensitivity and accuracy of the detection system. At the same time, the concentration of H_2_O_2_, the ratio and concentration of AuNCs and Cu^2+^, and the pH, temperature, and time of the reaction were optimized.

The feasibility study of AuNCs-Cu^2+^ as a probe for PPi detection includes both the selectivity of the AuNCs-Cu^2+^ system for PPi and the determination of PPi concentration in artificial urine. The selectivity of the AuNCs-Cu^2+^ system for PPi was evaluated by measuring the absorbance of the system at 452 nm with samples containing 20 μM of other common anions (ATP, AMP, Cl^−^, F^−^, I^−^, S^2−^, HCO_3_^−^, SO_4_^2−^, BrO_3_^−^, IO_3_^−^, ClO_3_^−^, and SiO_3_^2−^). The detection of the PPi concentration in artificial urine was used for the feasibility study of the AuNCs-Cu^2+^ system. Artificial urine samples were configured according to the literature [3], including CO(NH_2_)_2_ (310 mM), NaCl (55 mM), KCl (67 mM), Na_2_HPO_4_ (19.8 mM), Na_2_SO_4_ (29.6 mM), C_4_H_7_N_3_O (9.8 mM), MgSO_4_ (3.2 mM), and CaSO_4_ (2.6 mM). The PPi samples were prepared with diluted artificial urine (diluted 10-fold with BR, pH = 4.0).

The detection of the PPi concentration in human urine was used for the practical application of the AuNCs-Cu^2+^ system. Fresh human urine samples were obtained from three healthy volunteers throughout the day in a 24-h period and were stored in a refrigerator at 4 °C. The human urine was centrifuged at 2000 rpm for 15 min, and the clear supernatant was poured into another test tube. The PPi samples were prepared with diluted human urine (diluted 100-fold with BR, pH = 4.0). The detailed experimental conditions were as follows: 75 μL of AuNCs-Cu^2+^ (3 mg·mL^−1^), 10 μL of PPi (27 μM), 30 μL of H_2_O_2_ (3 mM), and 9 μL of TMB (10 mg·mL^−1^) were mixed with 776 μL of 100-fold diluted human urine solution (pH 4.0). The reaction time and temperature were 30 min and 40 °C, respectively. The subsequent steps were the same as the colorimetric assay of PPi described above.

### 2.4. Fluorescence Assay of PPi

The fluorescence assay of PPi was based on the change in the fluorescence signal of AuNCs-Cu^2+^. Before the test, the samples (AuNCs-Cu^2+^ with a final concentration of 1 µM Cu^2+^ and 0.05 mg·L^−1^ AuNCs and PPi with different concentrations) were mixed with BR buffer and incubated at 40 °C for 20 min. A fluorescence spectrometer (Edinburgh FS-5, UK) was then used to record the fluorescence response of AuNCs-Cu^2+^ to PPi at 675 nm (λ_ex_ = 400 nm).

## 3. Results

### 3.1. Principle of Detection for PPi

**Figure 1:**
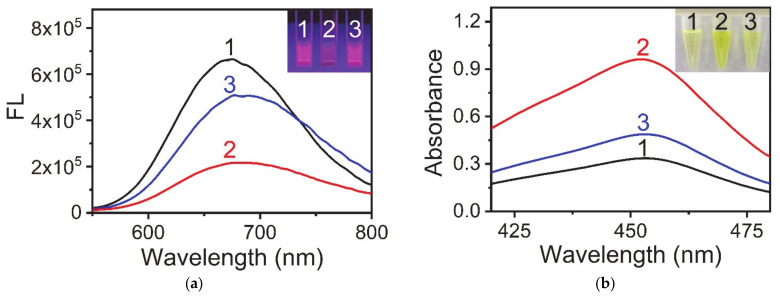
Interaction between AuNCs, Cu^2+^ ions and PPi. (**a**) Fluorescence emission spectra and photographs of samples under UV light (inset) in different reaction systems. (1) AuNCs, (2) AuNCs and Cu^2+^ ions, and (3) AuNCs, Cu^2+^ ions, and PPi. (**b**) UV-vis absorption spectra and photographs of samples under visible light (inset) in different reaction systems. (1) AuNCs, TMB, and H_2_O_2_; (2) AuNCs, Cu^2+^ ions, TMB, and H_2_O_2_; (3) AuNCs, Cu^2+^ ions, PPi, TMB, and H_2_O_2_. Reaction conditions: (**a**) AuNCs, 0.25 mg·mL^−1^; Cu^2+^, 10 μM; PPi, 100 μM; and BR buffer (pH 4.0); (**b**) AuNCs, 0.25 mg·mL^−1^; Cu^2+^, 10 μM; PPi, 10 μM; TMB, 0.1 mg·mL^−1^; H_2_O_2_, 100 μM; and BR buffer (pH 4.0).

**Figure 2:**
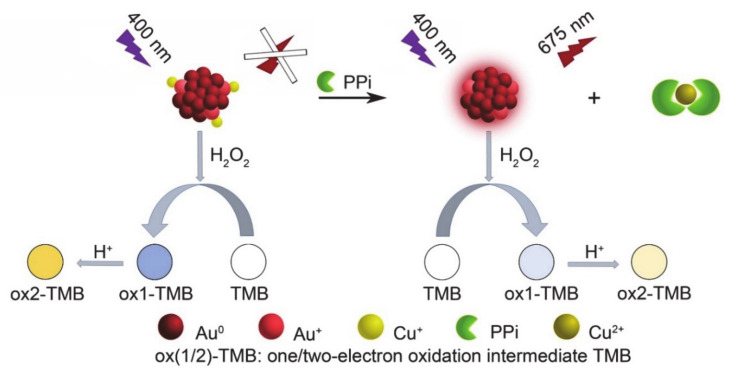
Schematic illustration showing the fluorescence and colorimetric methods of the PPi assay based on AuNCs-Cu^2+^.

Many studies have proven that AuNCs can be used as fluorescent probes for the detection of Cu^2+^ and PPi [27,28]. Figure 1a shows that the AuNCs synthesized using keratin as a template have the same fluorescence response properties. Pure AuNCs showed a strong fluorescence signal at 675 nm (Line 1), but the addition of Cu^2+^ weakened its fluorescence signal (Line 2), and the addition of PPi restored the fluorescence signal (Line 3). This phenomenon was considered to be due to the interaction between PPi and Cu^2+^ being stronger than that between Cu^2+^ and AuNCs. Our previous research showed that Cu^2+^ can enhance the catalytic activity of AuNCs. Combining the above studies on the interaction between AuNCs, Cu^2+^, and PPi, we tried to develop a colorimetric method for PPi detection using AuNCs as sensors. A schematic diagram of the detection method is shown in Figure 2, and the experimental results are shown in Figure 1b. Additionally, the catalytic activity of AuNCs could be assessed through a TMB color change [29,30]. Under acidic conditions, the more yellow the solution was, the stronger was the catalytic ability of AuNCs. As shown in Figure 1b, pure AuNCs showed a lower catalytic ability (Line 1), and the presence of Cu^2+^ greatly improved the catalytic ability of AuNCs (Line 2). However, when PPi was added into the system, the enhanced catalytic ability of AuNCs, which was due to the complex with Cu^2+^, was mostly lost (Line 3). The above experimental results showed that the detection of PPi with the AuNCs-Cu^2+^ catalytic system was feasible.

To prove that the loss of the catalytic ability of AuNCs was due to the complexation of Cu^2+^ on AuNCs by PPi, we carried out the following verification experiments. Three reaction solutions (system 1: AuNCs, TMB, and H_2_O_2_; system 2: AuNCs-Cu^2+^, TMB, and H_2_O_2_; and system 3: AuNCs-Cu^2+^, PPi, TMB, and H_2_O_2_.) were incubated at 40 °C for 30 min and then centrifuged. Finally, Na_2_S was added to the supernatant. The solubility product constant (pK_sp_) of CuS was 35.2 and the complexation constant (lgK_w_) of [Cu(P_2_O_7_)_2_]^6−^ was 12.45 [31]. Therefore, S^2−^ could convert the free Cu^2+^ and Cu-PPi complexes ([Cu(P_2_O_7_)_2_]^6−^) in the solution into black CuS precipitates. As shown in Figure S1, a black precipitate was produced in system 3, which was visible to the naked eye, while clear and transparent solutions were formed in systems 1 and 2. The above experimental results proved that the Cu^2+^ that complexed on the AuNCs was very stable in the absence of PPi and would not be free in solution (system 2). However, PPi competed with Cu^2+^ on the AuNCs and existed in the solution as a complex compound ([Cu(P_2_O_7_)_2_]^6−^).

### 3.2. Quantitative Detection of PPi Based on AuNCs-Cu^2^

Our previous research showed that the catalytic ability of AuNCs had a linear relationship with the concentration of Cu^2+^ [25]. Therefore, we believe that there should be a linear relationship between the reduction of the catalytic ability of AuNCs-Cu^2+^ and the concentration of PPi. To improve the sensitivity of PPi detection, the concentration of each component in the AuNC-Cu^2+^/TMB/H_2_O_2_ system, as well as the reaction conditions, were optimized.

The optimization of the reactant concentrations in the AuNC-Cu^2+^/TMB/H_2_O_2_ system is shown in Figure S2. Conditions represented by the blue lines were the optimized conditions. The selection principle of the optimization conditions was that the response of the system changed as much as possible in the case of a low concentration of PPi. When the concentration of H_2_O_2_ was 100 μM and 500 μM, the response trends of the system to the concentration of PPi were almost the same. To save cost, 100 μM H_2_O_2_ was chosen as the reaction condition. In summary, the optimal concentration of each reactant was 10 μM Cu^2+^ corresponding to 0.25 mg·mL^−1^ AuNCs and 100 μM H_2_O_2_. The optimization of the reaction conditions of pH and temperature in the AuNC-Cu^2+^/TMB/H_2_O_2_ system is shown in Figure S3. Before and after the addition of PPi, the absorbance intensity of the system was changed as much as possible, and pH 4 and 40 °C were identified as optimal reaction conditions. Additionally, the optimal reaction time was 30 min (Figure S4).

**Figure 3:**
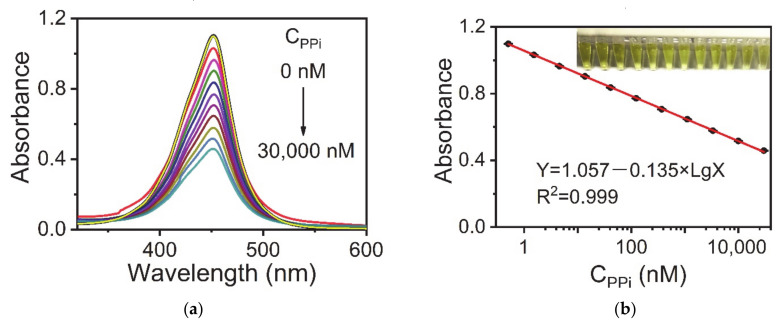
(**a**) UV-vis spectra of the AuNC-Cu^2+^/TMB/H_2_O_2_ system upon the addition of different concentrations of PPi (0–30,000 nM) corresponding to (**b**) absorbance intensities versus the logarithmic concentrations of PPi. Reaction conditions: AuNCs-Cu^2+^, 0.25 mg·mL^−1^; TMB, 0.1 mg·mL^−1^; H_2_O_2_, 100 μM; and BR buffer (pH 4.0). The error bars represent the standard deviation of three measurements.

Figure 3 shows the calibration curve for PPi detection under the optimal conditions. The fitting formula obtained was: Y = 1.057 − 0.135 Lg X (R^2^ = 0.999), where Y is the absorbance of the sample solution at 452 nm and X is the concentration of PPi. The standard error of the intercept was 0.004 while the standard error of the slope was 0.001. The linear range of PPi was from 0.51 to 30,000 nM. The LOD was 0.49 nM, which was estimated from the blank mean minus three times the standard deviation. Notably, the LOD of this method was three orders of magnitude lower than that of the fluorescence method using AuNCs-Cu^2+^ as a probe. In Figure S5 the linear range of PPi was only from 1 μM to 300 μM using AuNCs as fluorescent probes. The LOD was 0.82 μM, which was estimated from the blank mean plus three times the standard deviation.

### 3.3. Feasibility of AuNCs-Cu^2+^ Probe for PPi Detection in Practical Applications

**Figure 4:**
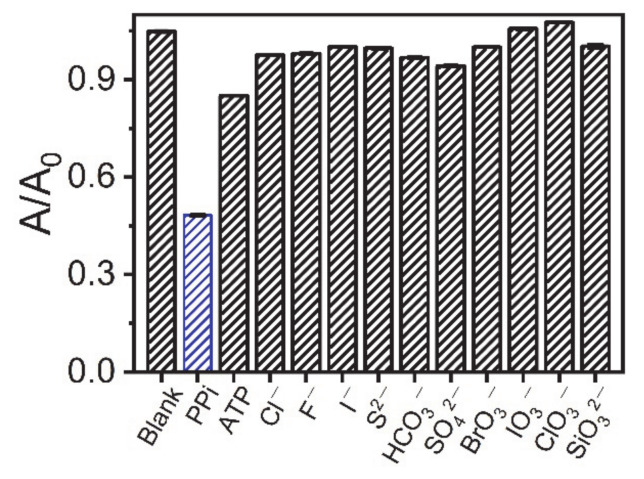
Determination of various interfering ions (20 µM additions).

In addition to the sensitivity of the AuNC-Cu^2+^ system, the feasibility of the AuNC-Cu^2+^ system for the analysis of biological samples using this method was further explored. First, we looked at the sensor selectivity of this method. Different anions (ATP, Cl^−^, F^−^, I^−^, S^2−^, HCO_3_^−^, SO_4_^2−^, BrO_3_^−^, IO_3_^−^, ClO_3_^−^, and SiO_3_^2−^) were tested. Instead of PPi, the above anions were added to the reaction solution at a concentration of 20 μM. The results in Figure 4 demonstrate that the effect of most anions on AuNCs-Cu^2+^ was negligible relative to PPi. However, the presence of ATP, which is a phosphate-related anion, had a slight effect on PPi detection. The presence of PPi reduced the absorbance of the AuNCs-Cu^2+^system at 452 nm by 53.9%, while ATP decreased the absorbance by 18.1%. The current observations were acceptable because PPi was formed via ATP hydrolysis.

**Figure 5:**
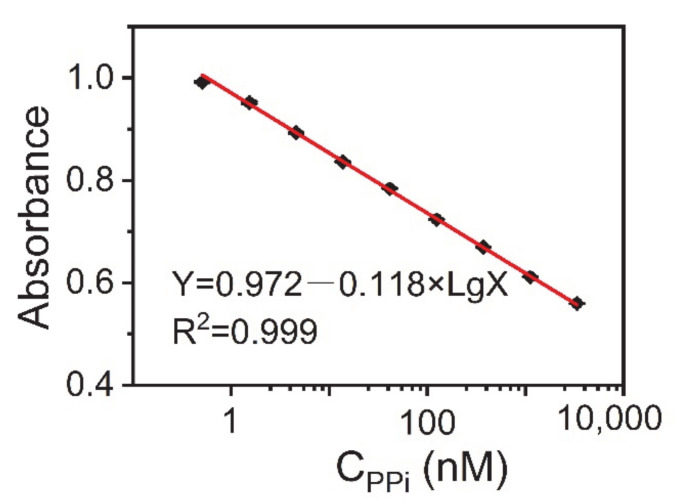
Calibration curve between the absorbance at 452 nm against the logarithmic concentration of PPi in artificial urine samples. Reaction conditions: AuNCs-Cu^2+^, 0.25 mg·mL^−1^; TMB, 0.1 mg·mL^−1^; and H_2_O_2_, 100 μM. The error bars represent the standard deviation of three measurements.

Second, we determined the level of PPi in the artificial urine samples. Figure 5 shows the calibration curve for PPi detection. The fitting formula obtained was: Y = 0.972 − 0.118 Lg X (R^2^ = 0.999), where Y is the absorbance of the sample solution at 452 nm and X is the concentration of PPi. The standard error of the intercept was 0.003, while the standard error of the slope was 0.001. It was deemed satisfactory that this method had the same high sensitivity in artificial urine as the standard solution. The linear range of PPi was from 0.51 to 30,000 nM. The LOD was 0.09 nM, which was estimated from the blank mean minus three times the standard deviation. Based on the above results, we believe that the AuNC-Cu^2+^ system can be used for the detection of PPi in biological samples.

### 3.4. Detection of PPi in Urine Samples

As listed in Table 1, the detection range of our method (reaching five orders of magnitude) was better than those of most previously reported methods. At the same time, the recovery reaction proved that the experiment had good reliability. The PPi level in urine is associated with urolithiasis and kidney-related diseases. The concentration range of PPi in normal human urine is 1.8 × 10^−5^–11 × 10^−5^ M [32,33,34]. The AuNC-Cu^2+^ sensing system was applied for determination of PPi in human urine according to the procedures described in Section 2. The results are listed in Table 2. Using the standard addition method, the recoveries of spiked PPi ranged from 94.95% to 100.79% with RSDs lower than 0.06%. All these data indicate that the colorimetric assay by the AuNC-Cu^2+^ system has great potential for the detection of PPi in biological samples.

## 4. Conclusions

In this work, a very sensitive colorimetric method for the quantification of PPi by the AuNCs-Cu^2+^ system was demonstrated. Compared with only AuNCs, AuNCs-Cu^2+^ showed high catalytic activity in the TMB-H_2_O_2_ system. However, in the presence of PPi, the catalytic activity of AuNCs-Cu^2+^ decreased since the affinity between Cu^2+^ and PPi was higher than that between Cu^2+^ and AuNCs. As a result, PPi can be easily quantified with the AuNCs-Cu^2+^ system. Under optimal conditions, this method had a very wide linear range (0.51 to 30,000 nM), reaching five orders of magnitude, and had excellent sensitivity with an LOD of 0.49 nM. This method also had satisfactory recoveries (between 94.95% and 100.79%), indicating that this method shows promise for use in the quantitative determination of PPi in other biological samples.

## Figures and Tables

**Table 1:** Performance comparison of the detection methods for detection of PPi.

Method	Probe	Linear Range (μM)	LOD (nM)	Ref.
DFM	AuNPs	0–0.49	1.49	[3]
EL	Cu^2+^/Cys/Au	0.1–10,000	10	[35]
FL	Ag@SiO_2_-AuNCs-Cu^2+^	0.5–60	78.7	[23]
FL	BSA-AuNCs-Cu^2+^	0.16–78.1	83	[24]
FL	AuNCs-Cu^2+^	1–300	820	This work
COL	CDs	0–100	4.29	[36]
COL	SiO_2_-PDA-Cu^2+^	0.1–300	60	[10]
COL	AuNCs-Cu^2+^	0.05–30	0.49	This work

Abbreviations: EL, electrochemistry; FL, fluorescence; COL, colorimetry; CDs, carbon dots, CuNPs, Cu nanoparticles; SiO_2_-PDA-Cu^2+^, silica-polydopamine hybrids-Cu^2+^; Ref., references.

**Table 2:** Recovery results for PPi detection in human urine samples.

Sample	Original Amount (nM)	Added Amount (nM)	Founded Amount (nM)	RSD (%) (*n* = 3)	Recovery (%)
1	401.24	300	703.62	0.03	100.79
2	480.03	300	775.12	0.05	98.37
3	540.56	300	825.40	0.06	94.95

Note: The human urine samples were diluted 100-fold with buffer (BR, pH = 4.0).

## Data Availability

Not applicable.

## Supplementary Materials

The following are available online at https://www.mdpi.com/article/10.3390/s21165538/s1, Figure S1: PPi competes for Cu^2+^ ions on AuNCs through a complexation reaction, Figure S2: Optimization of the concentration of reactants in AuNC-Cu^2+^/TMB/H_2_O_2_ system, Figure S3: Optimization of the reaction conditions (a) pH and (b) temperature-dependent absorbance intensities of the AuNCs-Cu^2+^ (0.25 mg·mL^−1^) catalytic system before and after the addition of 10 μM PPi, Figure S4: Optimization of reaction time, reaction conditions, Figure S5: (a) Fluorescence emission spectra of AuNCs-Cu^2+^ upon the addition of different concentrations of PPi (0–300 μM, λex = 400 nm), corresponding to (b) fluorescence recovery efficiencies versus the logarithmic concentrations of PPi, Figure S6: Calibration curve between the absorbance at 452 nm against the logarithmic concentration of PPi in artificial urine samples.
