# Supplementary material for: Copper (II) Ion-Modified Gold Nanoclusters as Peroxidase Mimetics for the Colorimetric Detection of Pyrophosphate
Source: Sensors (Basel). 2021 Aug 17;21(16):5538. doi: 10.3390/s21165538 (PMC8400922; doi:10.3390/s21165538)
Supplement: Supplementary file 1 [file sensors-21-05538-s001.zip › sensors-1319375-supplementary.pdf]

sensors

MDPI

Copper (II) ion modified gold nanoclusters as peroxidase mimetic for colorimetric detection of pyrophosphate

Yunjing Shi[1], Jinjie Wang[1*], Kun Mu[1], Suqin Liu[1], Guang Yang[2], Min Zhang[1] and Jingxia Yang[1].

[1] College o f Chemistry and Chemical Engineering, Shanghai University of Engineering Science, 333 Longteng Road, Shanghai 201620, China; m040118166@sues.edu.cn (Y.S.); mkofficial@163.com (K.M.); m040119510@sues.edu.cn (S.L.); zhangmin@sues.edu.cn (M.Z.); jxyang@sues.edu.cn (J.Y.)

[2] College of Chemistry, Chemical Engineering and Biotechnology, Donghua University, 2999 North Renmin Road, Shanghai 201620, China; gyang@dhu.edu.cn

* Correspondence: jinjiewang@sues.edu.cn; Tel.: +86-21-6779-1221

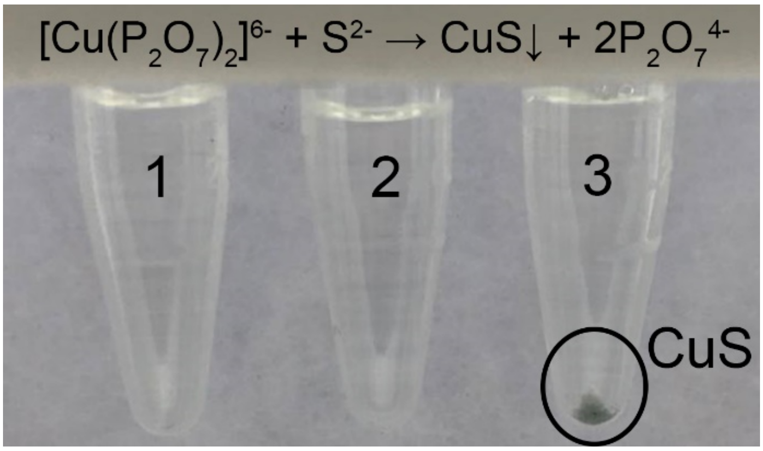

Figure S1. PPi competes for Cu^{2+} ions on AuNCs through a complexation reaction. (1) AuNCs, TMB and H_2O_2; (2) AuNCs-Cu^{2+}, TMB and H_2O_2; (3) AuNCs-Cu^{2+}, PPi, TMB and H_2O_2. The reaction solutions were incubated at 40 °C for 30 min, and then centrifuged. Finally, Na_2S was added to the supernatant. Reaction conditions: AuNCs, 0.25 mg·mL^{-1}; Cu^{2+}, 10 μM; PPi, 10 μM; TMB, 0.1 mg·mL^{-1}; H_2O_2, 100 μM; BR buffer (pH 4.0); Na_2S, 0.05 M.

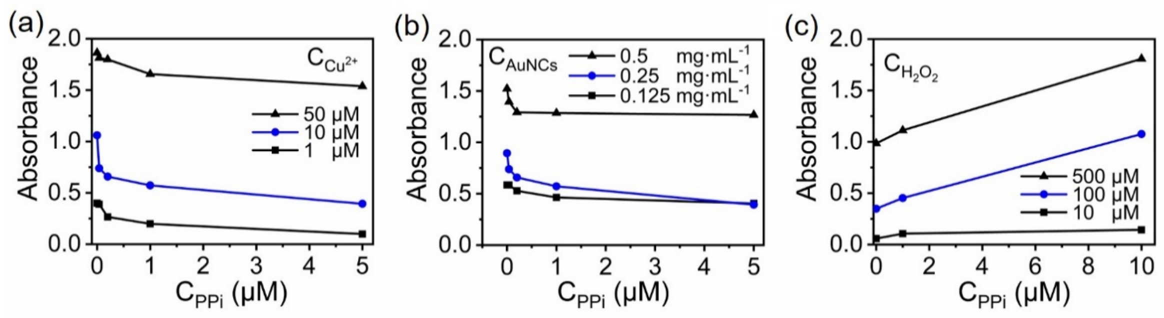

Figure S2. Optimization of the concentration of reactants in AuNC-Cu^{2+}/TMB/H_2O_2 system. (**a**) Cu^{2+}; (**b**) AuNCs; (**c**) H_2O_2. Reaction conditions: AuNCs, 0.25 mg·mL^{-1} (except b); Cu^{2+}, 10 μM copper ions corresponding to 0.25 mg·mL^{-1} AuNCs (except a); H_2O_2, 100 μM (except c); TMB, 0.1 mg·mL^{-1}. The blue line is the optimization condition.

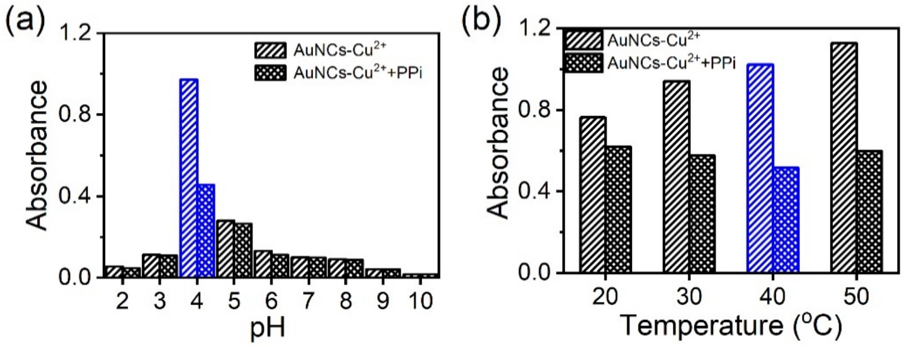

Figure S3. Optimization of the reaction conditions (**a**) pH and (**b**) temperature dependent absorbance intensities of the AuNCs-Cu^{2+} (0.25 mg·mL^{-1}) catalytic system before and after the addition of 10 μM PPi.

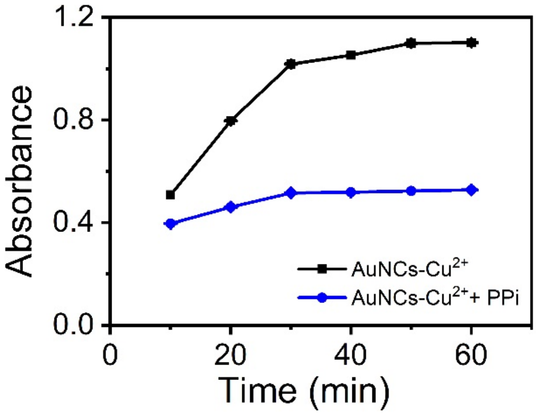

Figure S4. Optimization of reaction time. Reaction conditions: AuNCs-Cu^{2+}, 0.25 mg·mL^{-1}; H_2O_2, 100 μM; TMB, 0.1 mg·mL^{-1}; PPi, 10 μM. The error bars represent the standard deviation of three measurements.

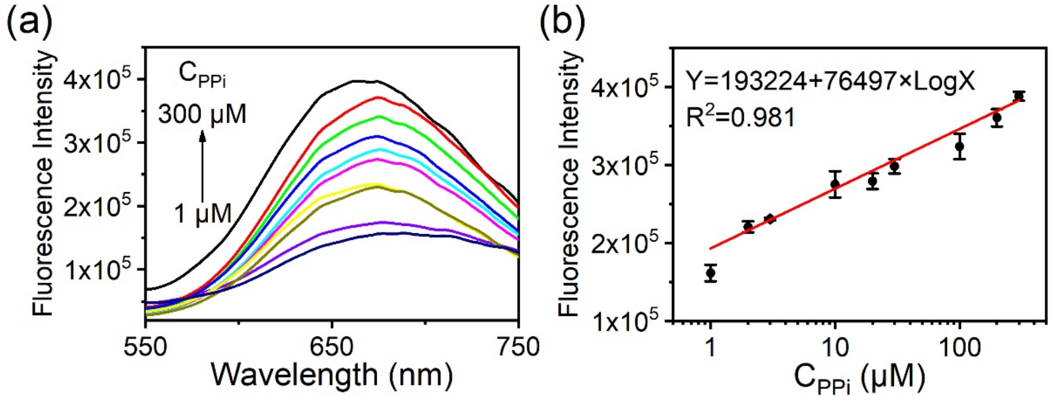

Figure S5. (**a**) Fluorescence emission spectra of AuNCs-Cu^{2+} upon the addition of different concentrations of PPi (1-300 μM, λ_{ex}=400 nm), corresponding to (**b**) fluorescence recovery efficiencies versus the logarithmic concentrations of PPi. Reaction conditions: AuNCs-Cu^{2+}, 0.05 mg·mL^{-1}; BR buffer (pH 4.0). The error bars represent the standard deviation of three measurements.
